# Trends in Hospital Admissions for Psychoactive Substance Intoxication Among Children, Adolescents, and Young Adults in Slovenia, 2013–2023

**DOI:** 10.3390/jcm15062112

**Published:** 2026-03-10

**Authors:** Barbara Lovrecic, Mateja Rok Simon, Mercedes Lovrecic

**Affiliations:** National Institute of Public Health, Trubarjeva 2, 1000 Ljubljana, Sloveniamateja.rok.simon@nijz.si (M.R.S.)

**Keywords:** trends of hospitalization, acute intoxication by psychoactive substances, new psychoactive substances, public health, psychiatric implication

## Abstract

**Background/Objectives**: Intoxication by psychoactive substances (PASs) in children, adolescents, and young adults is a growing public health concern, with evolving patterns of use and hospital presentation. This study aimed to analyze the trends in hospital admissions for PASs among children and youth aged 10 to 24 years in Slovenia during the 2013–2023 period. **Methods**: We performed a retrospective observational study on patients discharged after hospitalization due to poisoning by PASs, according to ICD 10 AM. We considered four groups: children (aged 10–14), adolescents (aged 15–19) and young adults (20–21 and 22–24 years old). Annual hospitalization rates were stratified by sex, age group, and PAS category. The joinpoint regression model was used to estimate the average annual percentage change (AAPC) and annual percentage change (APC) response time trend. **Results**: Of those hospitalized, 52% were male and 65% were adolescents, followed by children (13%). A statistically significant decrease in alcohol-related hospitalizations was observed in the 10–14 and 15–19 age groups for both sexes in the period 2013–2023, while a statistically significant increasing trend was observed for alcohol in 22–24-year-old males during the period 2019–2023, and in multiple drug/other/unspecified PASs in the 15–19 age group in the period 2015–2023. **Conclusions**: Slovenia has some peculiarities in the abuse of PASs. Sex reversal phenomena are present already among children (especially for alcohol), and there are shifting risks in polydrug use in adolescents and emerging threats, as well as an increase in sedative or hypnotic poisoning in female adolescents since 2017.

## 1. Introduction

Intoxication with psychoactive substances (PASs) is a significant and growing concern among adolescents, leading to a range of health, behavioral, and social problems. Alcohol and other illicit drugs, but also new psychoactive substances (NPSs), including designer drugs, are the most common causes of intoxication, often resulting in hospital admissions. Alcohol is the most frequently used PAS among adolescents, especially those aged 15–16, while other substances (including NPSs and medications) are more common in older teens [1,2,3].

Alcohol use creates risks of around 230 different identified health outcomes taking the form of disease or injury, including infectious and non-communicable diseases (according to the International Statistical Classification of Diseases and Related Health Problems—10th Revision—ICD-10) [4,5]. In children and adolescents, alcohol intoxication usually proves to be a symptom of a broader problem requiring further investigations to identify potential problems that need special attention. In adolescents, alcohol use is likely to be episodic and to involve larger volumes (often taking the form of binge drinking) than those of adults. Adolescence is a time of vulnerability and adjustment [6], accompanied by various risky behaviors, including experimentation with the initial stage of alcohol consumption, when impulse control is still relatively immature [7]. Adolescent drinking raises the likelihood of engaging in risky behaviors and developing mental health problems later in life [8,9,10]. Early initiation into drinking, with the consequent risk of contracting alcohol use disorder, could be related to manifestations of psychopathology [11,12].

There is a rising trend in the use of NPSs, such as synthetic cathinones, cannabinoids, and designer benzodiazepines. These substances are often used by boys and are difficult to detect with standard drug tests [13,14,15,16]. Hospital admissions for intoxication by PASs among children, adolescents, and young adults have been increasing in recent years, with repeated emergency department (ED) visits for PAS concerns rising significantly, especially among young adults and males. Polysubstance use, as well as the use of opioids, cocaine, and stimulants, is strongly associated with repeated ED visits, while cannabis and alcohol remain the most commonly involved PASs in single-visit cases [17,18].

Alcohol is the most common PAS leading to hospital treatment among young people, but the use of NPS has been rising, particularly among adolescents and young adults. Boys are more likely to use designer drugs, while girls more frequently use stimulants such as alcohol and cigarettes. Adolescents are more likely to present with cannabis and benzodiazepine intoxication, while young adults more often present with cocaine, amphetamines, and other substances. Polydrug use is a significant concern, especially among males aged 10–19 years [1,16,17,19,20].

The clinical symptoms of intoxication vary by age and PAS. Intoxication can cause a wide range of symptoms, including disturbed or loss of consciousness, drowsiness, visual disturbances, hypothermia, gait disturbances, aggression, sedation, confusion, respiratory depression, hypotension, bradycardia, muscle stiffness, and liver damage [1,13,14,16].

Alcohol intoxication is often the most severe, while NPSs can cause unpredictable and atypical symptoms due to varying compositions [1,14,16]. Adolescents more often present with diminished consciousness, while young adults are more likely to experience anxiety, palpitations, and chest pain. In small children, unintentional intoxication is more common, whereas adolescents typically engage in intentional, episodic use. The most severe intoxications are associated with alcohol, and repeated hospitalizations are increasing annually. Most cases resolve with supportive care, including fluid therapy and sedation, within 48 h, and severe outcomes such as intensive care unit (ICU) admission or death are rare [1,16,17,19,21].

Diagnosis and management of intoxication in pediatric and adolescent populations are challenging due to the variability in clinical presentation and the difficulty in detecting certain substances, such as NPSs, with standard toxicology tests. Systematic evaluation and supportive management are the mainstays of treatment, as specific antidotes are rarely available. Temporary observation units in emergency departments are effective for management most cases, and hospitalization is often not required unless complications arise [16,17,21,22].

Risk factors for hospital-treated intoxication include male sex, co-occurring mental health disorders, difficult socio-economic situations, and a history of alcohol or other PAS use. The central nervous system of children and adolescents may be more vulnerable to the effects of PASs, increasing the risk of adverse outcomes. The COVID-19 pandemic and associated lockdowns led to a decrease in hospitalizations for intoxication, particularly among young adults, likely due to reduced social and recreational use [1,16,18,20,21].

To obtain important baseline data that would provide a scientific basis for the prevention and management of hospitalization due to PAS poisoning, we conducted this study. Examining trends in hospitalizations related to PAS use over the past decade provides a unique opportunity to assess the overall societal burden of these hospitalizations. In this study, we analyzed trends in hospital admissions due to PAS exposure in children (10–14 years), adolescents (15–19 years), individuals aged 20–21 years, and individuals aged 22–24 years in Slovenia during the period 2013–2023. We considered hospital admissions due to alcohol, opioids, cannabinoids, sedatives or hypnotics, cocaine, other psychostimulants, hallucinogens, inhalants, and polydrug use according to the ICD-10 diagnostic description.

## 2. Materials and Methods

### 2.1. Research Design

A retrospective observational study on psychoactive substance poisoning in children, adolescents and young adults was conducted.

### 2.2. Data Sources

Data on hospital discharges (hospitalizations) from the National Hospital Health Care Statistics Database (NHHCSD) (ref: SBO) over the time period 2013–2023 was used. Data reporting on all admissions to hospitals including admissions due to poisoning is mandatory and nationwide. The database manager is the National Institute of Public Health of Slovenia.

Hospital discharge cases were defined as first hospitalizations, while readmissions were excluded. The main discharge diagnosis was defined using ICD-10 AM (ver.6) [5] codes and classified into one of the following nine types of intoxication: alcohol: F10.0 or T51.0–T51.9; opioids: F11.0 or T40.0–T40.4, T40.6; cannabinoids: F12.0 or T40.7; sedatives or hypnotics: F13.0 or T42.3–T42.4; cocaine: F14.0 or T40.5; other stimulants with abuse potential: F15.0 or T43.6; hallucinogens: F16.0 or T40.8; inhalants: F18.0 or T41.0, T52, T53, T59.8, or T65.8; multiple drug use/other/unspecified: F19.0 or T50.9.

In the National Hospital Health Care Statistics Database, each hospitalization can only have one primary discharge diagnosis, which in this case is coded either T (toxic effects) or F (acute intoxication).

### 2.3. Participant Characteristics

The study population included 2129 hospital discharge cases due to psychoactive substance poisoning in children (10–14 years), adolescents (15–19 years) and young adults (20–21 and 22–24 years) (Table 1). A slightly more than half of them were males (52.3%, N = 1114), while 47.7% (N = 1015) were females.

### 2.4. Ethical Issues

The data on hospital discharges at the national level was acquired in an aggregated (by year of discharge, main diagnosis, sex and age groups) and anonymized form; there-fore, no permission from the Republic of Slovenia Ethics Committee was required for this study.

### 2.5. Statistical Analysis

Hospitalization data related to psychoactive substance intoxication was analyzed for the period 2013–2023. Annual hospitalization rates were stratified by sex (total population, females, and males), age group (10–14, 15–19, 20–21, and 22–24 years), and substance category (alcohol, cannabinoids, opioids, sedatives or hypnotics, cocaine, other stimulants, hallucinogens, inhalants, and multiple/other/unspecified substance). The annual age- or age-and-sex-specific crude hospitalization rate for each psychoactive substance was calculated by dividing the number of hospitalizations recorded in each year by the corresponding number of inhabitants as of 1 July each year (data acquired from the Statistical Office of the Republic of Slovenia) and expressed per 100,000 inhabitants.

Temporal trends were assessed using joinpoint regression analysis by the Joinpoint Regression Program, V 4.7.0.0 (Statistical Research and Applications Branch, National Cancer Institute, Rockville, Maryland, MD, USA). The calendar year was specified as the independent variable, and log-transformed hospitalization rates were modeled to estimate changes in trends over time. Model selection was performed using a data-driven weighted Bayesian Information Criterion, allowing the identification of statistically significant joinpoints while accounting for the limited length of the time series. Segment-specific annual percent changes (APCs) and average annual percent changes (AAPCs) over the entire study period were estimated, together with 95% confidence intervals. All statistical tests were two-sided, and statistical significance was assessed at the 0.05 level. Joinpoint regression could not be performed for strata with zero values in one or more years due to the use of log-transformed rates; such strata were excluded from trend modeling. Graphical outputs were generated for all analyzable cohorts.

## 3. Results

### 3.1. Age- and Sex-Specific Patterns of Intoxication with Psychoactive Substances

The average age-specific hospitalization rate due to poisoning with all PASs was the highest in the 15–19 age group. Men had higher hospitalization rates than women in all age groups except children (10–14 years), but different age- and sex-specific patterns of intoxication with different substances are shown. In Table 2, average annual hospitalization rates (per 100,000) during the 2013–2023 period due to intoxication with various PASs are presented. Alcohol poisoning had the highest hospitalization rate in all age groups and in both sexes. High hospitalization rates were also observed for sedative or hypnotic intoxication in females aged 15–19 years, and due to multiple/other/unspecified drugs in females aged 15–19 years and males aged 20–21 years.

### 3.2. Substance-Specific Trends

During the 2013–2023 period, annual age- and sex-specific hospitalization rates due to intoxication with alcohol have decreased in all age groups. In contrast, hospitalization rates in children have increased for intoxication with cannabinoids (only in males, from 0.00/100,000 in 2013 to 3.39/100,000 in 2023), other psychostimulants (only in females, from 0.00/100,000 to 1.80/100,000), inhalants (only in females, from 0.00/100,000 to 5.41/100,000) and multiple/other/unspecified drugs (males: from 0.00/100,000 to 1.69/100,000; females: from 2.27/100,000 to 5.41/100,000). Hospitalization rates in adolescents have increased for intoxication with opioids (only in males, from 2.00/100,000 to 7.71/100,000), cocaine (only in females, from 0.00/100,000 to 2.03/100,000), other psychostimulants (only in females, from 0.00/100,000 to 12.18/100,000), hallucinogens (males: from 0.00/100,000 to 1.93/100,000; females: from 0.00/100,000 to 2.03/100,000), inhalants (only in females, from 2.11/100,000 to 6.09/100,000) and multiple/other/unspecified drugs (males: from 5.99/100,000 to 15.42/100,000; females: from 10.56/100,000 to 18.27/100,000). Hospitalization rates in adults aged 20–21 years have increased for intoxication with sedatives or hypnotics (males: from 0.00/100,000 to 4.77/100,000; females: from 9.83/100,000 to 16.14/100,000) and multiple/other/unspecified drugs (males: from 13.85/100,000 to 23.83/100,000; females: from 4.92/100,000 to 10.76/100,000). Lastly, hospitalization rates in adults aged 22–24 years have increased for intoxication with cocaine (only in females, from 0.00/100,000 to 10.23/100,000). In Table 3. annual age-specific hospitalization rates (per 100.000) due to intoxication with various PASs during the 2013–2023 period are presented.

Joinpoint regression analysis was successfully performed for 17 out of 108 predefined cohorts, stratified by sex, age group, and substance category. Analysis demonstrated consistent and statistically significant declines in alcohol-related hospitalizations in the 10–14 and 15–19 age groups, across both sexes. Joinpoints were detected in alcohol-related hospitalization rates in the 20–21 age group, and in males aged 20–21 and 22–24 years. Analysis revealed that hospitalization rates showed a statistically significant increasing trend in males aged 22–24 years during the period 2019–2023.

**Figure 1:**
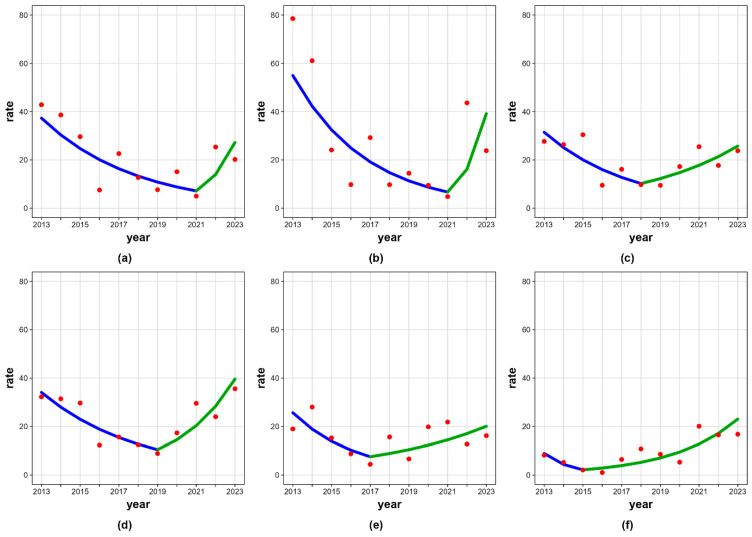
Joinpoint regression in hospitalization rates (per 100,000) due to intoxication with various psychoactive substances by age and gender, Slovenia, 2013–2023. (**a**) Alcohol, all aged 20–21 years; (**b**) alcohol, males aged 20–21 years; (**c**) alcohol, all aged 22–24 years; (**d**) alcohol, males aged 22–24 years; (**e**) sedatives or hypnotics, females aged 15–19 years; (**f**) multiple/other/unspecified drugs, all aged 15–19 years.

Trends for other psychoactive substances were heterogeneous, frequently non-significant, reflecting lower event counts and greater temporal variability. Joinpoints were detected in hospital rates due to intoxication with sedatives or hypnotics in females aged 15–19 years, and due to intoxication with multiple drug/other/unspecified in the 15–19 age group. Analysis revealed that the latter showed a statistically significant increasing trend during the period 2015–2023. Detailed joinpoint regression results stratified by sex, age group, and substance category are presented in Table 4 and Figure 1.

## 4. Discussion

Among all hospitalized persons in our case, males made up slightly over half, and the largest share of all hospitalizations occurred among those aged 15–19 (65%), followed by those aged 10–14 years (13%). Alcohol poisoning was the most common cause of hospitalization throughout the observation period in all age groups. In 2023, by far the highest rate of hospitalization due to alcohol poisoning was recorded among adolescents (15–19 years), with the rate being 1.45 times higher in females than in males. In 2023, for all age groups, the second-highest rate of hospitalizations was due to multiple drug use/other PAS/unspecified drugs. This was followed by volatile solvent poisoning in children, tranquilizers and hypnotics in adolescents and those aged 20 to 21, and cocaine in those aged 22 to 24 years.

Regarding hospitalization rates for polydrug/other PAS/unspecified drugs in 2023, these were higher in all age groups than in 2013, while the opposite was true for alcohol. The highest rate for polydrug/other/unspecified drugs was recorded in 2023 for 20–21-year-olds, followed by adolescents; rates were higher for females in children and adolescents, while they were higher for males among the 20–21 and 22–24 age groups. In 2023 adolescents also had the highest hospitalization rate for opioids, which was higher for males, while cannabinoids, cocaine, other stimulants and hallucinogen poisoning were higher for females. Sedative or hypnotic poisoning was more frequent among females in all age groups; the highest rate was recorded in 20–21-year-olds and adolescents. In 2023, the highest rate of hospitalization due to inhalant poisoning was recorded in adolescents and children, and in both cases the rate was higher in females.

In the period 2013–2023, among all PASs, alcohol poisoning was the most common in all age groups (highest in adolescents and particularly high in girls aged 10–14), followed by poisoning with sedatives or hypnotics (which was consistently higher in females in all age groups and highest in adolescents), and in third place was poisoning with multiple drugs/other PAS/unspecified drugs (highest rates among males in the 20–21 age group and highest rates among females in adolescents and children, with the largest sex differences).

A statistically significant decrease in alcohol-related hospitalizations was observed in the 10–14 and 15–19 age groups for both sexes during the 2013–2023 period. A statistically significant increasing trend was observed in alcohol-related hospitalizations in males aged 22–24 years during the period 2019–2023, and in multiple drug/other/unspecified-related hospitalizations in the 15–19 age group in the period 2015–2023. An increasing trend was found in alcohol-related hospitalizations among all aged 20–21 (2021–2023), males aged 20–21 years (2021–2023), and all aged 22–24 (2018–2023), and in hospitalizations due to sedative or hypnotic intoxication among females aged 15–19 (2017–2023), but the differences were not statistically significant. The results of our research are consistent with the European School Survey Project on Alcohol and Other Drugs (ESPAD) and Health Behaviour in School-aged Children (HBSC) survey data.

According to the ESPAD, a nationwide research survey which collects data across around 40 European countries on the use of PASs and other forms of risky behavior among 15–16-year-old school students, in the period 1995–2024, a significant long-term decline in traditional PAS use across Europe has been shown, with lifetime illicit drug use dropping from 17% in 2019 to 14% in 2024. According to ESPAD averages for 15–16-year-olds, alcohol remains the most prevalent substance despite a 23% decline in binge drinking since 1995. While boys traditionally show higher lifetime prevalence for most substances, a “narrowing sex gap” is evident as alcohol use stabilizes among girls while continuing to decline among boys. Slovenia presents distinct challenges within this framework: its prevalence of high-risk cannabis use (5.9%) and NPSs (6%) are among the highest in Europe, with the latter being double the ESPAD average (3%). ESPAD also assesses high-risk cannabis use through the Cannabis Abuse Screening Test (CAST), applied to students who reported using cannabis in the past year. Slovenia (with Czechia) represents the maximum recorded risk level among 15–16-year-olds regarding high-risk cannabis. Furthermore, early initiation is a critical concern in Slovenia, where 5% of students report using inhalants by age 13 or younger, more than double the European average of 2.2%. For pharmaceutical use, a rising trend is noted in the non-medical use of tranquilizers and sedatives (8.5% average) across all regions [23].

The HBSC study is a large school-based survey carried out every four years in collaboration with the WHO Regional Office for Europe. The most recent HBSC survey (2021/2022) was conducted across 44 countries and regions in Europe, Central Asia and Canada. Substance use remains a crucial public health problem among adolescents. Adolescents’ current use of all substances increased sharply with age; substance use was generally higher in boys than girls at age 11, while the sex gap tended to narrow or disappear from age 13. Data from the 2022 survey confirms wide variability in substance use among countries and regions and underscores that PAS use escalates sharply with age, with alcohol use (consuming alcohol at least once in the past 30 days) jumping from a low prevalence of 8% of boys and 5% girls at age 11 to 57% lifetime use among 15-year-olds, with girls (38%) now exceeding boys (36%) in current consumption. A “sex reversal” trend is increasingly visible by age 15, as girls now equal or surpass boys in current alcohol consumption (38% vs. 36%) and binge drinking. Slovenia consistently ranks above HBSC averages for alcohol, with Slovenian 15-year-olds showing significantly higher rates of lifetime drinking (60% vs. 57% of the European average), repeated drunkenness (binge drinking/inebriation) and significantly higher current use of alcohol (use in last 30 days) for 11- and 13-year-olds. By age 15 in Slovenia, the sex gap for both alcohol and cannabis almost entirely disappears, mirroring international trends but manifesting at higher absolute prevalence levels. While European cannabis lifetime use slightly declined to 12% by 2022, Slovenia’s prevalence remains robustly higher than its international peers [24]. Our results are also consistent with those of other authors, but the specificity of Slovenian characteristics must be taken into account. According to several authors across pediatric and youth cohorts, intoxication and PAS use differ clearly by age. In European ED data on recreational drug toxicity (2013–2016), those less than 20 years old most often presented with cannabis [25]. Hospitalized children show a peak in mid–late adolescence due to alcohol or other PASs [26,27,28]. In a Polish pediatric cohort (0–18 years, 2016–2018), alcohol poisonings were significantly more frequent at 13–16 years, and mixed-drug poisonings at 17–18 years [1]. A Croatian 2016–2021 study (0–18 years) found medicaments were the leading cause of intoxication (41%), followed by alcohol (33%); non-alcohol drug intoxications were common in 14–18-year-olds, often as suicide attempts, especially in girls [28].

A comparative synthesis framework from the literature, with broader age groups, showed that based on the observed period 2013–2023 and the main sample, several conclusions can be drawn from the literature. In early adolescence (around 10–14 years), emergency room visits are increasing, but the proportion is still small; hospitalizations were mainly due to alcohol and cannabis [1,28,29]. In middle and late adolescence (15–19 years), the main pattern is a high burden of intoxication and mixed trends, with a peak in alcohol and mixed drug intoxication; however, in Europe, there has been a decline in alcohol consumption, while in Brazil, all intoxications have decreased slightly [1,21,28,30]. In young adults (approximately 18–24/29 years), the main pattern is the highest absolute amount, with recreational use in nightlife (alcohol, stimulants, and cannabis). There is a sharp decline associated with COVID-19 lockdowns, followed by a recovery with increasing cannabis use [21,25,29,31,32].

Regarding youth trends over time, in France (2014–2020), hospital stays for acute PAS intoxication (opiates, cocaine, benzodiazepines, psychostimulants, alcohol, and cannabis) decreased during COVID-19 lockdowns in 18–29-year-olds (up to −39%); alcohol intoxication admissions fell, while benzodiazepine- and cannabis-related stays rose over the period [21]. In a large U.S. youth ED system (2018–2023), PAS-related ED visits among 12–21-year-olds increased from 2.8% to 3.4% of all visits; 79% of visits were by 18–21 year olds, but rates rose significantly in 12–14- and 15–17-year-olds. Alcohol remained the most common, but cannabis-related visits nearly doubled (from 18% to 35%) across all age groups [29]. Brazilian national data (2017–2022) on hospitalizations for mental and behavioral disorders due to alcohol/other PASs in adolescents showed a decreasing hospitalization rate overall, from 16.2 to 13.7 per 100,000, with the steepest decline in 15–19-year-old males [30]. U.S. national data for emerging adults aged 18–24 years (1998–2014) showed increasing alcohol/other drug overdose hospitalizations (especially alcohol plus opioids) despite declines in alcohol-impaired driving and some injury deaths [31].

Emergency admissions for adolescent substance intoxication show notable trends over recent years, with alcohol remaining the most common cause and a general increase in cases involving other substances. Patterns vary by age, sex, and socio-demographic factors, and there are important implications for prevention and intervention. According to Trefan et al., there has been a general decrease in alcohol-related emergency hospital admissions among adolescents in some regions (Wales, 2006–2011), but alcohol remains the leading cause of intoxication-related admissions in this age group [33]. Females in younger age groups are more likely to be admitted than males, but this reverses in older adolescents [33]. While alcohol dominates, there is a growing concern about admissions related to other PASs, including cannabis and prescription drugs [34]. In France, pediatric admissions for cocaine intoxication increased eightfold over 11 years, with a rise in severe cases [35]. Regarding sex differences, females are more likely to be admitted for alcohol intoxication in early adolescence, while males predominate in late adolescence and for other substances [33,36]. Regarding the severity of PAS-related emergency hospital admissions among adolescents, most cases are mild to moderate, but there is an increase in severe presentations, especially with substances like cocaine [35,37].

Since 2010, hospital and ED admissions for PAS intoxication in adolescents and young adults appear to have increased overall, especially for cannabis, alcohol (in specific windows), and NPSs, though high-quality global time-series focused specifically on pediatric intoxication remain limited. Evidence since 2010 indicates rising emergency visits and hospital encounters for acute intoxication with alcohol, cannabis and NPSs among adolescents and young adults, with notable spikes around policy or social shifts (e.g., cannabis legalization and COVID-19 periods). Emergency and hospital encounters for illicit drugs occur several times more often among people who use drugs than in the general population, and many users are in late adolescence/young adulthood [37,38]. Reviews focusing on acute intoxication management highlight a growing burden of ED presentations from cannabis, cocaine, opiates and synthetic drugs in younger patients, with most having short stays but significant resource use [22]. Liberalization policies, such as legalization/decriminalization, have been accompanied by increased cannabis-related ED visits and hospitalizations in adolescents and young adults despite mixed effects on overall prevalence [22,39,40]. Multiple reviews describe a marked expansion of NPSs since 2010 and frequent poisonings/ED visits among teens and young people with severe psychiatric symptoms (psychosis, agitation, and anxiety) and occasional fatalities [20,41,42]. In our case we were not able to calculate trends specifically for NPSs, which in our case are included in poisoning with multiple drugs/other PAS/unspecified drugs.

Most studies report overall declines in youth PAS use during COVID-19 pandemic restrictions, but subgroups with poor prior mental health showed increased use, including illicit drugs [43]. After Italy’s lockdown, severe alcohol intoxications in 13–24-year-olds jumped from 0.9% to 11.3% of all ED visits, much higher than the same period in 2019 [44]. In our case, an impact analysis of the COVID-19 period (2020–2021) was not performed due to the small number of cases.

The data from our case highlights a critical shift in the Slovenian landscape. Regarding a “sex reversal” and “narrowing gap”, girls are catching up with or surpassing boys in PAS use [23,24]. In our case males had higher hospitalization rates than females in all age groups except children (10–14 years), while annual hospitalization rates for poisoning by sedatives or hypnotics prevailed in females in all age groups. PAS poisonings were higher in females: for children due to alcohol, inhalants and multiple drugs/other/unspecified, in adolescents (15–19 years) for opioids, cocaine, inhalants, and multiple drugs/other/unspecified, in those aged 20–21 years for hallucinogens, and in those aged 21–24 years for cocaine. While alcohol-related hospitalizations have decreased, hospitalizations for multiple drugs/other/unspecified PASs have increased significantly, representing shifting risks. Among those aged 15 to 19, the significant increase in hospitalizations for multiple drugs/other/unspecified PASs since 2015 and the increase in sedative or hypnotic poisoning in females since 2017 represent emerging threats. Special attention should be paid to NPSs, as multiple drug/other/unspecified PAS-related hospitalizations include, among others, NPS poisoning. The “sex reversal” is not merely statistical; it is reflected in emergency medical data in clinical response and acute care management. The data from our study indicates that annual hospitalization rates for PAS poisoning are now higher in females, especially among children and adolescents, for selected PASs. There is a need to prioritizing female poisoning risk in triage. As the gender gap in children in Slovenia is disappearing, emergency medical doctors may lower the age limit for a thorough toxicological evaluation, especially in young girls who have non-specific neurological symptoms. As the use of pharmaceuticals (sedative or hypnotic) for non-medical purposes is increasing among girls, protocols need to be updated to include mandatory screening for polydrug use and underlying mental health crises during the initial triage of adolescents (psychosocial assessment). Special attention should be paid to the possibility of NPS presence. There is a need, in these cases, to shift from “drug testing” to “toxidrome management”. EDs can no longer rely on standard test screens, which often fail to detect these synthetic compounds. Clinical protocols require symptom-based treatment and should focus on treating the toxidrome (the set of symptoms) rather than the PAS. For example, extreme agitation, hyperthermia, and tachycardia are treated as a “stimulant toxidrome” (common with synthetic cathinones) regardless of a negative drug test. For suspected NPS poisoning, there is a need for an observation window. EDs should consider a 6–12 h observation period for suspected synthetics/polydrug use, especially since NPSs’ toxidrome can be life-threatening, even for patients who appear stable, as these substances have unpredictable and delayed effects. Emergency protocols in acute care settings need to be prepared for the increased number of females with alcohol and polydrug/stimulant intoxications.

From a strategic public health recommendation point of view, there is a need for priorities in gender-specific prevention, lowering the age of intervention and enhanced surveillance. In gender-specific prevention, the intervention programs must move away from male-centric models. There is an urgent need for prevention strategies that address the specific psychosocial drivers of binge drinking, medication misuse among adolescent girls and multiple drug/other/unspecified PAS use among adolescents, including NPSs. Given that sex differences diminish among Slovenian children and adolescents, education must be implemented in primary school settings, as well as early screening for risky behaviors in primary health care and lowering the age of intervention. To address the NPS threat, Slovenia should invest in “sentinel” hospital-based surveillance. For enhanced surveillance, using appropriate methods (e.g., high-resolution mass spectrometry) for retrospective analysis of poisoning cases should allow public health officials to identify new substances entering the market in real time.

‘Gender-shifting’ trend in PAS use calls for new prevention and policy recommendations. The convergence and, in some cases, reversal of PAS use between sexes require a shift in public health strategy. Traditionally, substance use prevention was designed with a “male-centric” risk profile in mind, focusing on externalizing behaviors and illicit drug use. There is a need to take into consideration gender-responsive prevention and intervention. Due to the high rates of binge drinking and inebriation among girls, children and adolescents, targeted awareness-raising is needed, and programs must now specifically address this topic. Given the rise in non-prescribed tranquilizer and sedative use increasing female hospitalization rates for poisoning, policies should take into consideration psychosocial focus and integrate mental health support (addressing anxiety and stress) directly into substance use prevention.

The strong points of this study include its collection of national data (all the hospitals in Slovenia reported their available data), making use of decades of routinely implemented information systems and the mandatory reporting of data as required by law, treatment within Slovenia’s national health system (in a hospital environment) and full implementation of the diagnostic process, independently of any self-reporting. A particular strength is the monitoring of the incidence of hospitalization, which provides an appraisal of the impact of PAS use on health care. Hospital discharge cases were defined as first hospitalizations, while readmissions were excluded (no repeated hospitalizations).

Studies about hospital treatment of children, adolescents and young adults for PAS-related poisoning usually include the most common PASs such as alcohol and other most used illicit drugs. In our case we included alcohol, opioids, cannabinoids, sedatives or hypnotics, cocaine, other psychostimulants, hallucinogens, inhalants, and polydrug use/unknown/and other PASs, which include NPSs. In addition, for each intoxication by PASs, we used a combination of ICD 10 AM codes, from the chapters on mental and behavioral disorders due to acute poisoning (F10–19; these include mental and behavioral disorders due to substance use ‘with intoxication’ in the title) and on toxic effects (selected codes, e.g., T40; these codes are part of a chapter that also represents poisoning, and could be used for overdose surveillance with ICD-10-CM coded discharge data), which allows us to capture a larger number of cases of PAS poisoning. In the National Hospital Health Care Statistics Database, each hospitalization can have only one primary diagnosis at discharge, coded with T (toxic effects) or F (acute intoxication), and overlap or double counting is excluded. As there has not been any official change in our admission policy, nor in methodological steps, we believe that our data shows the actual trends of hospital admissions due to intoxication by PASs in Slovenia.

## 5. Conclusions

In summary, intoxication by PASs in children, adolescents, and young adults is a growing public health concern, with evolving patterns of PAS use and hospital presentation. Multiple substance use is of extreme concern among children and adolescents. Intoxication by NPSs seems to also be included in intoxication by multiple drug use/other/unspecified drugs. NPSs in children, adolescents, and young adults pose growing challenges for emergency and psychiatric services, but direct pediatric NPS data is sparse; most information comes from mixed-age ED/forensic series and broad reviews. In pediatric emergencies, most intoxications (including NPSs when recognized) are managed with supportive care and treatment of complications, as specific antidotes are rare [17,38].

In cases of poisoning due to NPSs, the central nervous system and cardiological system are usually altered. In severe poisonings, various toxidromes are clinically manifested, where antidotes are usually not available and the maintenance of vital life functions is a matter of concern. Poisoning with NPSs should also be considered in the event of a sudden acute clinical picture with psychosis in children and adolescents who have an elevated body temperature and altered cardiac parameters [40,45,46,47,48,49,50,51].

The narrowing gender gap in PAS use is a clinical reality reflected in our hospital wards. Mental health and emergency medicine professionals should prepare for an increased number of female patients presenting with complex, acute intoxications involving highly potent synthetic substances and pharmaceutical cocktails.

## 6. Limitations

An inherent limitation of the ICD 10 codes used is the lack of precision and the risk of formulating errors. Within the category of hospitalization due to poisoning with multiple drugs/other/unspecified PASs, we cannot quantify which cases are separately due to multiple drug use or unknown substance poisoning or NPSs. Study limitations include insufficient data on the places and times when PAS consumption occurred, types and quantities of PASs or other concomitant drug use. The sources of PASs were unknown, and there was no specific information on prescribed medication. There was no information available on in-depth toxicology testing (toxicological analyses performed for, e.g., for NPSs). We were unable to trace data on the reasons and circumstances that had led to PAS intoxication or data about another concomitant psychopathology. The numbers of children, adolescents and young adults intoxicated by PASs are probably higher than those recorded here, as our database did not include cases with only minor signs of PAS intoxication or situations when the intoxicated person had not requested medical treatment (e.g., some patients were treated at home). The results can be applied to the population of persons hospitalized for PAS poisoning and are not representative of the entire population of PAS users, nor of the general population. Assessing consequences or finding details about further treatment(s) after hospital discharge was not feasible. During the lockdowns and the pandemic period, people avoided going to hospital because they feared contracting the virus. However, patients with acute PAS intoxication and without hospitalization were less likely to present with severe conditions. The observation period was relatively short, and a longer observation period could have allowed for more accurate monitoring of trends.

## Figures and Tables

**Table 1:** Sociodemographic characteristics of the sample (N = 2129).

	Men	Women	Total
	Number (%)	Number (%)	Number (%)
10–14 years	117 (5.5)	163 (7.7)	280 (13.2)
15–19 years	715 (33.6)	679 (31.9)	1394 (65.5)
20–21 years	133 (6.2)	69 (3.2)	202 (9.4)
22–24 years	149 (7.0)	104 (4.9)	253 (11.9)
Total	1114 (52.3)	1015 (47.7)	2129 (100.0)

**Table 2:** Average hospitalization rates (per 100,000) due to intoxication with various psychoactive substances by age and sex, and male-to-female ratio, Slovenia, 2013–2023 period.

	10–14 Years			15–19 Years			20–21 Years			22–24 Years		
Psychoactive Substance	M	F	M:F	M	F	M:F	M	F	M:F	M	F	M:F
Alcohol	17.46	20.72	0.84	103.78	92.41	1.12	28.06	12.73	2.20	22.66	15.78	1.43
Opioids	nc	0.60	/	2.37	2.39	0.99	3.90	1.83	2.13	3.49	0.88	3.97
Cannabinoids	1.19	0.76	1.57	7.22	5.73	1.26	2.56	1.82	1.41	2.42	1.40	1.73
Sedatives or hypnotics	0.93	4.26	0.22	5.01	15.33	0.33	3.05	7.99	0.38	2.18	6.35	0.34
Cocaine	0.17	nc	/	0.95	1.78	0.53	2.20	1.93	1.14	0.27	1.20	0.22
Other psychostimulants	nc	0.16	/	3.13	2.49	1.26	5.63	0.45	12.51	0.27	nc	/
Hallucinogens	nc	nc	/	1.49	0.38	3.92	0.43	0.46	0.93	0.53	nc	/
Inhalants	0.53	0.88	0.60	1.48	1.75	0.85	nc	0.48	/	0.51	0.26	1.96
Multiple/other/unspecified	0.64	3.50	0.18	6.99	11.55	0.61	11.74	4.81	2.44	7.50	4.43	1.69
Total	20.28	27.38	0.74	125.43	122.26	1.03	45.83	27.69	1.66	32.33	25.87	1.25

Notes: M: male; F: female; M:F = male-to-female ratio; nc: no cases; /: not applicable.

**Table 3:** Annual age-specific hospitalization rates (per 100,000) due to intoxication with various psychoactive substances by age group, Slovenia, 2013–2023 period.

Alcohol											
	**2013**	**2014**	**2015**	**2016**	**2017**	**2018**	**2019**	**2020**	**2021**	**2022**	**2023**
10–14 y	24.08	28.48	28.42	21.72	19.10	20.45	18.50	15.89	14.43	7.07	11.35
15–19 y	153.90	135.88	129.91	109.46	111.98	102.59	95.44	67.25	43.40	62.96	68.22
20–21 y	42.86	38.62	29.61	7.54	22.61	12.71	7.68	15.12	5.02	25.34	20.22
22–24 y	27.68	26.37	30.44	9.51	16.11	9.75	9.48	17.25	25.49	17.72	23.80
Opioids											
10–14 y	nc	nc	nc	2.17	nc	1.02	nc	nc	nc	nc	nc
15–19 y	1.03	1.05	2.11	4.25	nc	3.24	1.07	3.20	2.12	4.13	3.96
20–21 y	11.91	nc	4.93	5.02	nc	nc	nc	nc	nc	10.14	nc
22–24 y	2.77	2.93	6.09	1.59	3.22	1.63	1.58	1.57	nc	1.61	1.59
Cannabinoids											
10–14 y	nc	nc	1.09	1.09	1.06	3.07	nc	1.87	nc	0.88	1.75
15–19 y	9.23	9.41	8.45	8.50	14.00	1.08	4.29	5.34	3.18	3.10	4.94
20–21 y	7.14	7.24	7.40	nc	nc	nc	nc	nc	nc	nc	2.53
22–24 y	2.77	4.39	4.57	6.34	1.61	nc	nc	1.57	nc	nc	nc
Sedatives or hypnotics											
10–14 y	4.38	2.19	1.09	2.17	3.18	5.11	nc	1.87	0.90	6.18	0.87
15–19 y	12.31	15.68	9.51	6.38	6.46	9.72	4.29	11.74	16.94	7.22	9.89
20–21 y	4.76	9.66	9.87	2.51	5.02	7.63	5.12	nc	5.02	nc	10.11
22–24 y	6.92	4.39	7.61	7.93	nc	3.25	1.58	1.57	3.19	6.44	3.17
Cocaine											
10–14 y	nc	nc	nc	nc	nc	nc	0.97	nc	nc	nc	nc
15–19 y	nc	nc	1.06	1.06	3.23	3.24	2.14	nc	2.12	1.03	0.99
20–21 y	nc	2.41	nc	nc	2.51	12.71	nc	nc	2.51	2.53	nc
22–24 y	nc	1.46	nc	nc	nc	nc	1.58	nc	nc	nc	4.76
Other psychostimulants											
10–14 y	nc	nc	nc	nc	nc	nc	nc	nc	nc	nc	0.87
15–19 y	3.08	nc	1.06	3.19	3.23	1.08	4.29	nc	2.12	4.13	8.90
20–21 y	9.52	7.24	2.47	5.02	5.02	nc	2.56	nc	2.51	nc	nc
22–24 y	nc	1.46	1.52	nc	nc	nc	nc	nc	nc	nc	nc
Hallucinogens											
10–14 y	nc	nc	nc	nc	nc	nc	nc	nc	nc	nc	nc
15–19 y	nc	nc	nc	nc	1.08	2.16	nc	2.14	3.18	nc	1.98
20–21 y	nc	2.41	2.47	nc	nc	nc	nc	nc	nc	nc	nc
22–24 y	nc	nc	nc	nc	nc	nc	nc	1.57	1.59	nc	nc
Inhalants											
10–14 y	nc	nc	1.09	nc	1.06	nc	1.95	0.93	nc	nc	2.62
15–19 y	2.05	1.05	nc	3.19	2.15	1.08	3.22	1.07	nc	nc	3.96
20–21 y	nc	nc	nc	nc	nc	nc	nc	nc	2.51	nc	nc
22–24 y	2.77	nc	1.52	nc	nc	nc	nc	nc	nc	nc	nc
Multiple drug use/other/unspecified											
10–14 y	1.09	nc	3.28	1.09	1.06	2.05	3.89	0.93	5.41	nc	3.49
15–19 y	8.21	5.23	2.11	1.06	6.46	10.80	8.58	5.34	20.11	16.51	16.81
20–21 y	9.52	7.24	12.34	5.02	2.51	nc	17.92	nc	10.03	10.14	17.69
22–24 y	9.69	4.39	3.04	nc	8.06	3.25	6.32	6.27	7.97	8.05	9.52

Notes: y: years, nc: no cases.

**Table 4:** Temporal trends in hospitalization rates due to intoxication with various psychoactive substances by age and sex, Slovenia, 2013–2023 (joinpoint regression analysis).

	Sex	AAPC (%)	95% CI	*p* Value	Period Segment	APC (%)	95% CI	*p* Value
Alcohol								
10–14 years	All	−10.4 *	[−14.4, −5.9]	<0.001				
	F	−11.2 *	[−16.2, −5.8]	<0.001				
	M	−9.4 *	[−14.2, −4.2]	<0.001				
15–19 years	All	−10.0 *	[−13.6, −6.1]	<0.001				
	F	−7.8 *	[−13.5, −1.7]	0.016				
	M	−11.9 *	[−14.0, −9.9]	<0.001				
20–21 years	All	−3.1	[−21.3, 10.8]	0.502	2013–2021	−18.6	[−62.1, 89.4]	0.130
					2021–2023	95.0	[−28.1, 89.4]	0.263
	M	−3.3	[−22.1, 10.7]	0.516	2013–2021	−23.2	[−64.0, 18.6]	0.060
					2021–2023	141.9	[−18.8, 384.0]	0.158
22–24 years	All	−2.0	[−10.9, 7.8]	0.661	2013–2018	−20.2 *	[−53.4, −1.3]	0.042
					2018–2023	20.3	[−3.2, 110.8]	0.084
	F	−5.8	[−14.4, 3.6]	0.233				
	M	1.5	[−6.2, 8.6]	0.657	2013–2019	−17.9 *	[39.7, −7.0]	0.003
					2019–2023	39.5 *	[10.0, 127.3]	0.002
Cannabinoids								
15–19 years	All	−10.8	[−23.6, 4.6]	0.152				
	F	−10.7	[−21.1, 1.5]	0.088				
Sedatives or hypnotics								
15–19 years	All	−1.5	[−10.5, 8.7]	0.759				
	F	−2.4	[−15.6, 15.2]	0.755	2013–2017	−26.4	[−67.2, 54.2]	0.142
					2017–2023	17.7	[−47.1, 159.0]	0.169
	M	−2.8	[−11.5, 7.4]	0.572				
Multiple/other/ unspecified drugs								
15–19 years	All	9.9	[−1.6, 32.0]	0.097	2013–2015	−50.9	[−73.6, 23.6]	0.245
					2015–2023	34.4 *	[1.4, 146.4]	0.049

Notes: AAPC: Average annual percent change over the period 2013–2023, APC: Annual percent change, * indicates that the AAPC or APC is significantly different from zero at the alpha = 0.05 level.

## Data Availability

The raw data is unavailable due to privacy restrictions.

## Abbreviations

The following abbreviations are used in this manuscript:

PASs	Psychoactive substances
NPSs	New psychoactive substances
ED	Emergency department
ICU	Intensive care unit
ICD 10 AM	International Statistical Classification of Diseases and Related Health Problems, Tenth Revision, Australian Modification
WHO	World Health Organization
ESPAD	European School Survey Project on Alcohol and Other Drugs
HBSC	Health Behaviour in School-aged Children survey
